# The road to the electroreduction of CO_2_

**DOI:** 10.1038/s42004-023-01096-w

**Published:** 2024-03-05

**Authors:** Yuguang C. Li

**Affiliations:** Department of Chemistry, University at Buffalo, State University of New York, Buffalo, NY 14260 USA

The Guest Editor of *Communications Chemistry*’s Collection on electrocatalytic CO_2_ reduction discusses the importance of catalyst design, mechanistic investigation, and reactant transport for the electrochemical carbon dioxide reduction reaction (ECO_2_RR). The author also highlights some of the field’s pressing challenges in advancing ECO_2_RR further into industrial applications.

Addressing the pressing issue of global warming and the broad challenges climate change brings about necessitates innovative and sustainable solutions. One promising avenue is the ECO_2_RR: a chemical process with the potential to generate clean fuels and mitigate the harmful impacts of excessive CO_2_ in our atmosphere. This method offers not only a pathway to curb greenhouse gas emissions, but also presents an opportunity to transform a waste product into advanced fuels and chemicals, contributing to a carbon-neutral economy.

Despite active and ongoing global research efforts in ECO_2_RR, there is still much to be done to transition this technology from the laboratory to large-scale industrial application. While many successful electrocatalysts have been reported^1^, it is challenging for any catalyst to maintain good selectivity, activity and cost-effectiveness simultaneously. Recently, immense progress has been made in addressing key issues such as energy efficiency, single-pass utilization, product crossover, and carbonate formation^2,3^. Still, a clear winner electrochemical system, ready for industrial-level stability and scalability testing, has yet to be declared. Fundamentally, a much better understanding of the CO_2_ activation mechanism has been gained in the last decade, with a catalyst design strategy based on the Sabatier principle marking a milestone^4^. Beyond CO_2_ activation, a better fundamental understanding of the reaction pathway selectivity leading to C2+ products is still needed. To study these reaction mechanisms, new in situ or *operando* characterization tools are necessary to probe the complex nature of heterogeneous surfaces and electrochemical systems. While these challenges are daunting, the research community is thriving with the exploration in many different aspects of CO_2_ reduction. The transition of this technology to the industrial scale can only be achieved with a portfolio of diverse research directions. Rightfully so, the electrification of CO_2_ continues to gain popularity and competitiveness.

In this Collection on ECO_2_RR, we have gathered publications relating to CO_2_ electrochemical reduction in three major sections of CO_2_ research: understanding the reaction mechanism, material design for electrocatalysts, and electrochemical systems for devices.

## Understanding the mechanism

Understanding the fundamental mechanism of ECO_2_RR is in our opinion the most crucial direction for the research field. From a computational perspective, we can model reaction schemes that are otherwise challenging to study experimentally. Agarwal et al. developed a 2D model of the gas diffusion layer to probe the interplay between the applied potential and flow rate (10.1038/s42004-024-01122-5)^5^, identifying limitations in the mass transport through different types of gas diffusion layers. In order for models to be reliable, accurate methods need to be developed. At the ab initio level, Urrego-Ortiz et al. used different exchange correlation functionals to assess the gas phase error in computational electrocatalyst design for the co-electrolysis of CO_2_ and nitrogen (10.1038/s42004-023-00990-7)^6^, highlighting the importance of correcting free energy in computational electrocatalyst design.

Beyond computational studies, experimental characterization of the electrochemical surface is also critical for understanding ECO_2_RR mechanisms. The electrode surface is almost always heterogeneous and covered with solvent and electrolyte ions, adding complexity to the study of the electrochemical system. Mayer et al. reported a protocol using scanning electrochemical microscopy to study the CO_2_ reduction reaction on Sn/SnO_*x*_ surfaces (10.1038/s42004-020-00399-6)^7^. They were able to map the CO_2_ catalytic activity over different compositions and morphologies of the catalyst, allowing for fast and direct, localized surface analysis. Preikschas et al. reported an NMR spectroscopy protocol for high-throughput and accurate liquid products quantification (10.1038/s42004-023-00948-9)^8^. With this routine, they discovered previously unreported products obtained with a phosphate-derived Ni catalyst, and new mechanistic pathways for methane and long-chain product formation. Jovanovic et al. introduced an *operando* NMR technique to investigate the evolution of electrochemistry during CO_2_ electrolysis (10.1038/s42004-023-01065-3)^9^. They found that HCO_3_^−^ ions exist as ion pairs or free ions at different applied potentials. The equilibrium state between these two forms will affect the supply of CO_2_ for the reaction.

## Catalytic materials

A catalyst is the heart and soul of an electrocatalytic reaction. It needs to be selective towards a single desired product, while balancing high catalytic activity. Although Cu is the dominant choice of catalyst in the literature, it still has limitations in overcoming the Sabatier principle. Rabl et al. reported a metal-organic chalcogenolate assembly material (AgSePh and AgSPh) for CO_2_ reduction (10.1038/s42004-023-00843-3)^10^, marking the first time ECO_2_RR activity was reported for this material family. Beyond the active site element, the structure of the catalyst is also critical for catalyst performance. While Ag is a common catalyst for ECO_2_RR, Hoffmann et al. designed multi-scale, highly porous 3D Ag foam structures and demonstrated that CO_2_ reduction performance can be enhanced with a more homogeneous and denser foam structure (10.1038/s42004-023-00847-z)^11^. Furthermore, the second coordination sphere environment outside of the active site is also critical for ECO_2_RR performance. Fortunati et al. showed that imidazolium cations with different anions on the catalyst surface can regulate the ratio of cation and carbene on the catalyst’s surface (10.1038/s42004-023-00875-9)^12^, significantly influencing the selectivity of products and stability of the reaction. Ding et al. studied Au doping and ligand effects of Cu_25_, demonstrating that CO_2_ to formate activity is enhanced due to the electron distribution modulation caused by the catalyst design (10.1038/s42004-022-00779-0)^13^. These studies highlight the importance of not only the active site of the reaction but also the local environment where the reaction occurs.

Beyond direct CO_2_ reduction to chemical products, CO_2_ is also a co-reactant in more complex reactions—still achieving the goal of greenhouse gas mitigation, while generating valuable and more complex products. Anastasiadou et al. reported one of the most efficient catalysts to date for urea electrosynthesis: CuO_*x*_ZnO_*y*_ materials for the co-electrolysis of CO_2_ and nitrate to generate urea (10.1038/s42004-023-01001-5)^14^. Ju et al. reported carbonyl reduction with a single site M–N–C catalyst that could be applied to the electrocatalytic valorization of a biomass-derived feedstock (10.1038/s42004-023-01008-y)^15^.

## Electrochemical system design

The design of the electrochemical system is equally, if not more, important than the catalytic material. CO_2_ utilization, energy efficiency, single-pass ratio, products crossover, and carbonate mitigation are all topics currently being worked on. Samu et al. screened gas diffusion layers in CO_2_ electrolyzers to investigate the influence of the microporous layer, PTFE contents, layer structure and thickness on different device properties (10.1038/s42004-023-00836-2)^16^. Another important aspect of a CO_2_ electrolyzer is ion management by membrane separation and the resulting cathode design. A commentary provided by Chang et al. highlights recent advances in CO_2_ electrolyzers with bipolar membranes (BPM) (10.1038/s42004-022-00806-0)^17^. By incorporating a non-buffer catholyte layer into a BPM electrolyzer, the CO_2_ single-pass utilization (the ratio of carbon converted electrochemically to the total carbon input) can be enhanced due to the local chemical conversion of CO_3_^2−^ and protons from the BPM. These system-level engineering designs are crucial to improve the economic competitiveness of the electrochemical CO_2_ reduction reaction and bring this technology closer to industrial applications.

## Future outlook

Since the first report of CO_2_ reduction by Hori et al.^18^, ECO_2_RR technology has advanced significantly—we can now achieve catalytic current densities of up to ampere levels^19^, similar to those of water electrolyzers. Faradaic efficiencies for industrially relevant product generation, such as ethylene or ethanol, were reported to be as high as 80%, depending on the catalyst and the system^20,21^. We also have a better fundamental understanding of the CO_2_ activation step and the potential reaction pathways it follows. These advances are an undeniable indication that this technology could soon disrupt the chemical production industry.

However, we must also acknowledge that there is still work to be done to make ECO_2_RR technology truly economically competitive with traditional chemical production processes. From a catalyst design perspective, we should focus more on the local environment around the active site. This includes investigations into the coverage and potential dependence of intermediates during the reaction, adsorbate–adsorbate interactions, cation effects, and local electric field effects. From a system perspective, the cost of chemical production from CO_2_ is still not competitive. Therefore, improving the electrolyzer’s performance (energy efficiency >60% while maintaining >1 A/cm² current density for >1000 h) remains a high priority. Furthermore, more attention should be given to the single-pass utilization rate and the effects of mixed reactants with NO_*x*_/SO_*x*_ so that a CO_2_ electrolyzer can be directly coupled with industrial flue gases. With continued progress in research and development, we are confident that ECO_2_RR technology will fulfill its promise of delivering a greener chemical industry and positively impacting the global energy sector.
